# Biochemical isolation of myonuclei employed to define changes to the myonuclear proteome that occur with aging

**DOI:** 10.1111/acel.12604

**Published:** 2017-05-23

**Authors:** Alicia A. Cutler, Eric B. Dammer, Duc M. Doung, Nicholas T. Seyfried, Anita H. Corbett, Grace K. Pavlath

**Affiliations:** ^1^ Department of Pharmacology Emory University Atlanta GA 30322 USA; ^2^ Graduate Program in Biochemistry, Cell and Developmental Biology Emory University Atlanta GA 30322 USA; ^3^ Department of Biochemistry Emory University Atlanta GA 30322 USA; ^4^ Department of Biology Emory University Atlanta GA 30322 USA

**Keywords:** aging, brain, myonuclei, nuclear isolation, proteome, skeletal muscle

## Abstract

Skeletal muscle aging is accompanied by loss of muscle mass and strength. Examining changes in myonuclear proteins with age would provide insight into molecular processes which regulate these profound changes in muscle physiology. However, muscle tissue is highly adapted for contraction and thus comprised largely of contractile proteins making the nuclear proteins difficult to identify from whole muscle samples. By developing a method to purify myonuclei from whole skeletal muscle, we were able to collect myonuclei for analysis by flow cytometry, biochemistry, and mass spectrometry. Nuclear purification dramatically increased the number and intensity of nuclear proteins detected by mass spectrometry compared to whole tissue. We exploited this increased proteomic depth to investigate age‐related changes to the myonuclear proteome. Nuclear levels of 54 of 779 identified proteins (7%) changed significantly with age; these proteins were primarily involved in chromatin maintenance and RNA processing. To determine whether the changes we detected were specific to myonuclei or were common to nuclei of excitatory tissues, we compared aging in myonuclei to aging in brain nuclei. Although several of the same processes were affected by aging in both brain and muscle nuclei, the specific proteins involved in these alterations differed between the two tissues. Isolating myonuclei allowed a deeper view into the myonuclear proteome than previously possible facilitating identification of novel age‐related changes in skeletal muscle. Our technique will enable future studies into a heretofore underrepresented compartment of skeletal muscle.

## Introduction

Skeletal muscle, which is essential for critical processes such as movement, swallowing, and breathing, dynamically adapts to the body's needs by altering gene expression to accommodate physiological states such as muscle growth, regeneration, and aging. To date, investigations into molecular mechanisms driving shifts in gene expression have been largely candidate‐based. Examining global changes in myonuclear proteins would provide insight into molecular processes regulating large‐scale shifts in gene expression that accompany physiologic changes in muscle.

Aging leads to profound changes in skeletal muscle. Starting around age 30 and increasing after age 60, muscle mass and strength decline. The resulting frailty is associated with reduced mobility and greater risk of falls (Demontis *et al*., 2013). Global changes in RNA levels with age have been identified (Zahn *et al*., 2006; Kim *et al*., 2014; Su *et al*., 2015); however, large‐scale proteomic analysis has been limited to the most abundant proteins. While many cell types support skeletal muscle function, the tissue is comprised primarily of myofibers, large, multinucleated cells densely packed with contractile proteins necessary for muscle function. Over half of the total protein in a muscle cell is either actin or myosin (Deshmukh *et al*., 2015). This lack of heterogeneity in muscle tissue makes proteomic studies that span a large dynamic range of protein abundances technically challenging.

Limited information exists on the dynamic alterations of myonuclear protein levels during aging or other physiologic states. Myonuclear proteins are difficult to study with an unbiased approach because (i) nuclear proteins make up a small minority of proteins in skeletal muscle tissue due to the high abundance of contractile proteins, (ii) myonuclei are challenging to isolate because skeletal muscle is difficult to disrupt without damaging myonuclei, and (iii) dense debris co‐sediments with nuclei contaminating the nuclear fraction. In addition, muscle tissue is comprised of multiple cell types. Existing approaches do not provide sufficient enrichment to interrogate low abundance nuclear proteins, require large amounts of starting material, or necessitate genetic tagging of specific nuclei and have no mechanism of determining what proportion of isolated nuclei are myonuclei. An approach that would sufficiently enrich myonuclear proteins to allow unbiased interrogation would offer a new avenue to examine the molecular mechanisms regulating physiologic states in skeletal muscle.

By developing a method of purifying myonuclei from relatively small amounts of mouse muscle tissue, we were able to collect myonuclei for analysis by flow cytometry, biochemistry, and proteomics. Purification of myonuclei dramatically increased both the number and intensity of nuclear proteins detected compared to whole muscle tissue. Querying changes to the myonuclear proteome associated with aging revealed that nuclear levels of RNA processing proteins, nuclear transport proteins, and proteins regulating transcription increased with age. When compared to brain nuclei, many of the same processes were affected in both tissues with age, although the individual proteins that change differed between tissues. Our approach of isolating myonuclei will provide a useful tool for future studies of myonuclear changes in different physiologic states of skeletal muscle.

## Results

### Isolation of pure intact nuclei

**Figure 1:**
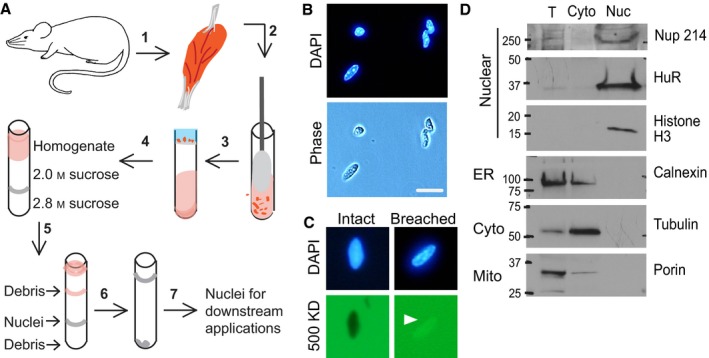
Workflow for isolating nuclei from skeletal muscle. (A) Workflow: 1) Skeletal muscle was dissected from mice and then 2) minced and homogenized. 3) The homogenate was filtered and nuclei pelleted at low speed. 4) The crude nuclear fraction was resuspended, layered over sucrose cushions, and 5) ultracentrifuged. 6) The nuclear fraction was collected, diluted, and pelleted at low speed. 7) The nuclear pellet was collected for downstream applications. (B) Purified nuclei were stained with DAPI and examined by microscopy. Nuclei were free from visible debris in phase and DAPI channels. Bar = 10 μm. (C) Nuclei were incubated with FITC‐conjugated 500 kDa dextran. Intact nuclear envelopes excluded the large dextran (93%), while envelopes breached during isolation (arrowhead) were permeable to the dextran (7%). Bar = 10 μm. (D) Nuclei (Nuc) were compared to total (T) and cytoplasmic (Cyto) fractions by immunoblotting. Nuclei were enriched for markers of the nuclear envelope (Nup 214), RNA‐binding proteins (HuR), and chromatin (Histone 3). Purified nuclei were also enriched in endoplasmic reticulum markers (ER) but depleted of cytoplasmic (Cyto) and mitochondrial (Mito) markers.

To facilitate biochemical and molecular analyses of myonuclei, we optimized a technique to isolate intact nuclei with high purity from single mouse muscles (Fig. 1A). To isolate nuclei, the muscle was gently homogenized. After preparation of a crude nuclear fraction by filtration and low‐speed centrifugation, a purified nuclear fraction was obtained by ultracentrifugation over a discontinuous two‐step sucrose gradient. Nuclei collect at the interface between the two sucrose cushions with cytoplasmic material above the first cushion and the denser myofibrils under the second cushion. The nuclear fraction was collected and analyzed by downstream methods. The average time required for preparation of a nuclear sample was 5.5 h. The average yield from a single mouse gastrocnemius (GA) muscle was 1 × 10^6^ nuclei, whereas pooling two GA and two rectus femoris (RF) muscles yielded 6 × 10^6^ nuclei and 8 μg of total protein.

The isolated nuclei were stained by 4′,6‐diamidino‐2‐phenylindole (DAPI) and were free of visible debris when examined by light microscopy (Fig. 1B). The nuclear envelope must remain intact to retain nuclear proteins for examination after purification. To determine whether the nuclear envelope was breached in the purification process, we challenged the nuclei with FITC‐conjugated 500 kDa dextran, which is excluded from nuclei with intact envelopes (D'Angelo *et al*., 2009). Nuclei were incubated with the dextran and analyzed by fluorescence microscopy. We found that 93% of isolated nuclei excluded the fluorescent dextran and thus had intact nuclear envelopes (Fig. 1C). To further examine whether soluble nuclear proteins were retained during isolation, and to determine the enrichment of nuclear proteins and depletion of proteins from other cellular organelles in the nuclear fractions, we immunoblotted total muscle tissue, as well as cytoplasmic and nuclear fractions (Fig. 1D, for full blots, see Fig. S1, Supporting information). Proteins from various nuclear compartments were enriched in the nuclear fraction including the nuclear envelope (Nup 214, a nuclear pore protein), the soluble fraction (RNA‐binding protein HuR), and chromatin (Histone H3). Immunoblotting for markers of mitochondrial, endoplasmic reticulum (ER), and cytoplasmic compartments, revealed that nuclear fractions were free of mitochondrial (porin) and cytoplasmic (tubulin) markers and depleted for the ER marker calnexin, although calnexin depletion was variable. Together, these results indicate that nuclei isolated using this method retain nuclear proteins, are depleted of other organelles, and have intact nuclear envelopes.

### Isolated nuclei are predominantly myonuclei

**Figure 2:**
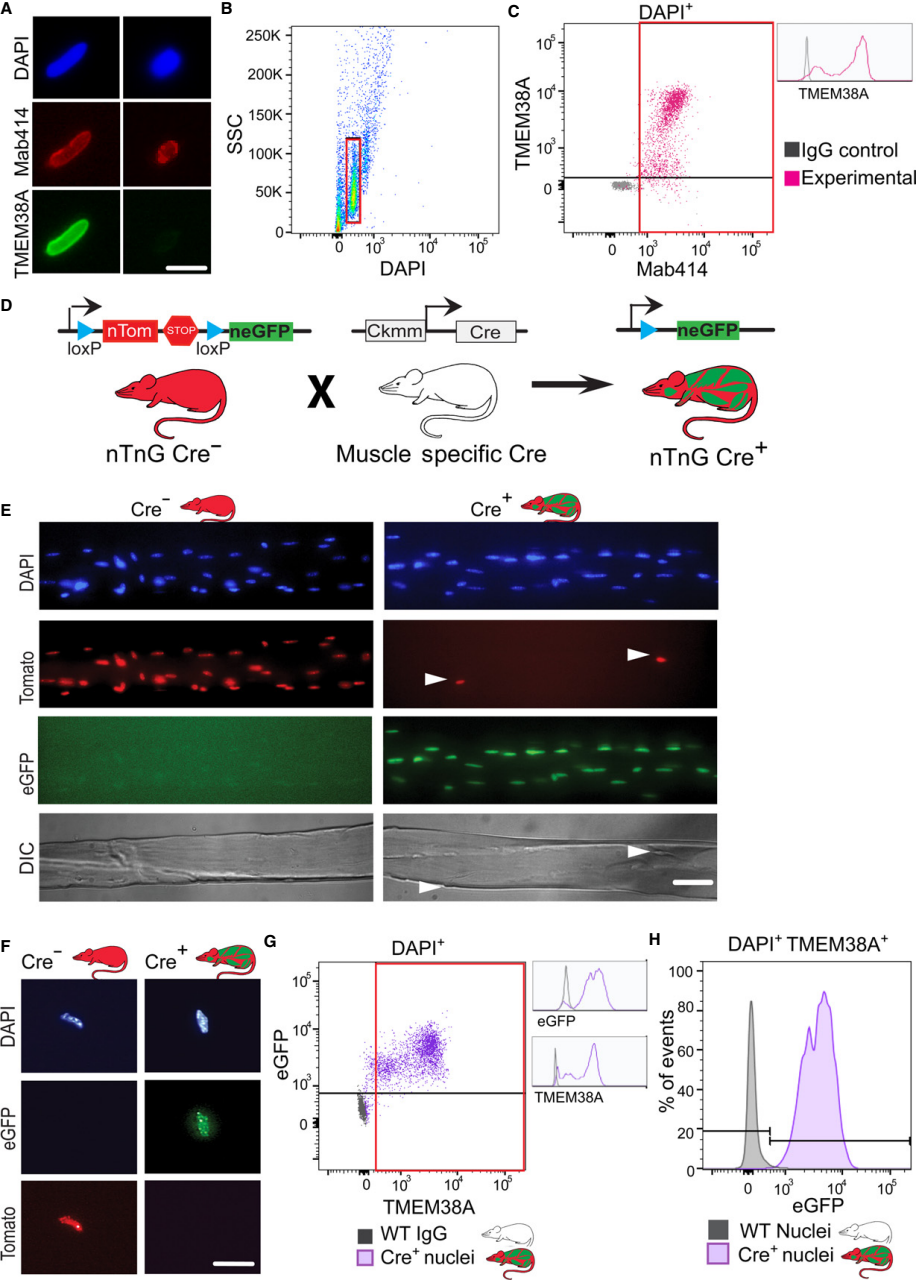
Isolated nuclei are predominately myonuclei. (A) Purified nuclei were stained with DAPI and immunostained with antinuclear pore complex antibody (Mab414) to label all nuclear envelopes and anti‐TMEM38A antibody to label myonuclei. Pictured are representative images of TMEM38A^+^ and TMEM38A^−^ nuclei. Bar = 10 μm. (B) Isolated nuclei were analyzed by flow cytometry by side scatter (SSC) and DAPI; intact DAPI ^+^ singlets were selected for further analysis (red gate). (C) IgG control immunostained nuclei (gray) were compared to experimental nuclei immunostained with Mab414 and TMEM38A antibodies (magenta). Myonuclei were defined to be double positive for Mab414 and TMEM38A (red gate); 96.4% ± 3.2 SE of Mab414^+^ nuclei were also positive for TMEM38A (*n* = 4). Bright and dim TMEM38A populations were distinct from control IgG immunostained nuclei (inset). (D) Transgenic mice with a cassette containing nuclear‐targeted tdTomato and eGFP reporter proteins (nTnG) were crossed with mice expressing Cre recombinase from a skeletal muscle‐specific promoter (Ckmm) to genetically label myonuclei and nonmyonuclei with distinct fluorescent markers in the offspring. All nuclei from wild‐type mice (WT) are nonfluorescent. All nuclei from nTnG ^+^ Ckmm Cre^−^ mice (Cre^−^) are fluorescent red. Myonuclei from nTnG ^+^ Ckmm Cre^+^ mice (Cre^+^) are fluorescent green, while nonmyonuclei are fluorescent red. (E) Single myofibers were isolated from Cre^−^ and Cre^+^ mice. Arrowheads indicate red nonmuscle cells on a myofiber containing green myonuclei. Bar = 50 μm. (F) Nuclei isolated from Cre^−^ and Cre^+^ mice retained their fluorescent label after isolation. Bar = 10 μm. (G) DAPI ^+^ nuclei isolated from a Cre^+^ mouse (purple) and a WT mouse (gray) were analyzed by TMEM38A and eGFP fluorescence; 85.5% ± 5.2 SE of nuclei from Cre^+^ mice were positive for both TMEM38A and eGFP (*n* = 4). Bright and dim TMEM populations were distinct from WT nuclei immunostained with control IgG (inset). TMEM38A^+^ nuclei were selected for further analysis (red gate). (H) TMEM38A^+^ nuclei were analyzed for eGFP fluorescence; 99.7% ± 0.03 SE of TMEM38A^+^ nuclei were eGFP ^+^, confirming that TMEM38A labels myonuclei.

As muscle tissue contains multiple cell types, we assessed what percentage of nuclei purified from muscle tissue were myonuclei by performing flow cytometry using myonuclear‐specific markers. TMEM38A is an outer nuclear envelope transmembrane protein expressed in excitatory cells with highest levels in myofibers (Bleunven *et al*., 2008). We took advantage of this property to use TMEM38A as a marker of myonuclei. Immunofluorescence analysis of isolated nuclei revealed that while all intact nuclei were labeled with a marker of the nuclear pore complex (mAb414), not all nuclei examined were positive for TMEM38A (Fig. 2A). Subsequently, we analyzed immunostained nuclei by flow cytometry. Singlet nuclei were identified by DAPI labeling and side scatter (SSC) and were gated for further analysis (Fig. 2B). To determine what percentage of intact nuclei was TMEM38A positive (TMEM38A^+^), we gated on mAb414‐positive (mAb414^+^) nuclei. Of this mAb414^+^ population of nuclei, 96.4% ± 3.2 SE were TMEM38A^+^ (Fig. 2C). While a bright and a dim population of TMEM38A^+^ nuclei were observed, both populations were distinct from nuclei labeled with control IgG (inset Fig. 2C). As TMEM38A localizes both to the nucleus and the sarcoplasmic reticulum (SR), the bright and dim populations of TMEM38A^+^ nuclei are likely the result of variable amounts of SR associated with isolated nuclei.

To confirm that the TMEM38A antibody specifically labels myonuclei, we took advantage of genetic labeling using the nTnG mouse model which contains a transgene with nuclear‐targeted tdTomato and eGFP (Prigge *et al*., 2013). As illustrated in Fig. 2D, the transgene is composed of a floxed nuclear localization signal (NLS)‐containing *tdTomato* gene followed by a stop codon; outside the floxed region is an NLS‐containing *eGFP* gene. Prior to Cre‐mediated recombination, the ubiquitously expressed tdTomato labels all nuclei. Upon recombination, the *tdTomato* gene and stop codons are excised and eGFP labels the nuclei of recombined cells. We bred these nTnG mice to mice expressing Cre recombinase under control of the muscle creatine kinase promoter (*Ckmm*) (Bruning *et al*., 1998). In the offspring of this cross, myonuclei are eGFP positive (eGFP^+^), while all other nuclei are tdTomato positive (tdTomato^+^). To confirm the specificity of myonuclear labeling in these offspring, we isolated single myofibers from GA muscles and visualized by fluorescence microscopy (Fig. 2E). All myonuclei within single myofibers were eGFP^+^ with only a few tdTomato^+^ cells on each myofiber. The fluorescent labels were retained during nuclear purification (Fig. 2F), making them reliable markers for subsequent flow cytometry analyses.

To compare the specificity of the two myonuclear markers, nuclei were isolated from the muscles of Cre^+^ nTnG mice and wild‐type mice, immunostained for TMEM38A or control IgG, and analyzed by flow cytometry (Fig. 2G). On average, 85.5% ± 5.2 SE of total nuclei were positive for both TMEM38A and eGFP, indicating that the vast majority of purified nuclei were myonuclei. As previously observed (Fig. 2C), both bright and dim populations of TMEM38A were distinct from the control IgG‐labeled wild‐type nuclei (inset Fig. 2G). To rule out the possibility that a subset of nonmyonuclei were labeled with TMEM38A, we further analyzed the TMEM38A^+^ nuclei and found that 99.7% ± 0.03 SE were eGFP^+^, indicating that TMEM38A labels myonuclei (Fig. 2H). Taken together, these results indicate that nuclei isolated by this method from skeletal muscle tissue are at least 85.5% ± 5.2 SE myonuclei.

### Increased depth of proteomic detection in myonuclear proteome

**Figure 3:**
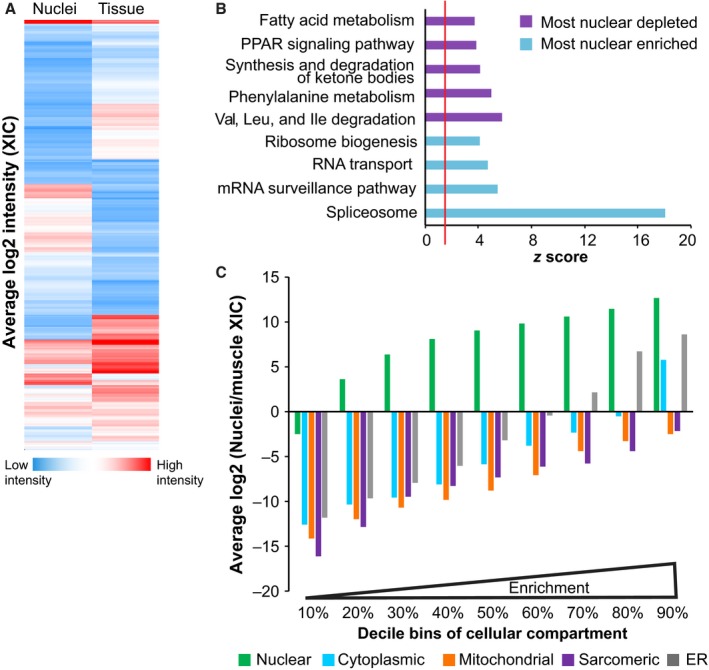
Nuclear proteins are enriched in purified nuclei. (A) The log2 of the mean extracted ion chromatogram (XIC) intensity of proteins from purified myonuclei, and whole muscle tissue samples are represented in a heat map: blue indicates low intensity (1.5 × 10^4^) and red indicates high intensity (1.5 × 10^10^) (*n* = 2 of each sample type). (B) The top KEGG pathways for the most depleted (purple) or most enriched (blue) proteins from isolated nuclei were plotted by z‐score significance. The red line indicates significance threshold (*z* = 1.96). (C) Proteins unique for nuclear, cytoplasmic, mitochondrial, sarcomeric, and endoplasmic reticular (ER) compartments were assigned by DAVID. Within each compartment, proteins were binned in deciles from the least enriched to the most enriched in nuclei compared to whole muscle tissue and plotted against the mean log 2 ratio of XIC in purified nuclei and whole muscle samples. This view reveals the consistency of enrichment and depletion within groups of proteins.

We next compared the proteomes of whole muscle tissue samples and isolated nuclear samples using mass spectrometry. Nuclei were purified from GA and RF muscles pooled from three mice. Of 1771 proteins detected in purified nuclear samples, 906 were not detected in whole muscle samples; similarly, 921 of 1786 proteins detected in the whole muscle samples were not detected in isolated myonuclei. We compared the average peptide LC peak intensity area for biological duplicates of nuclear and whole muscle samples and determined that nuclear samples were distinct from whole muscle samples (Fig. 3A). The most enriched proteins in purified nuclei clustered predominately to Kyoto Encyclopedia of Genes and Genomes (KEGG) pathways in RNA processing, while the most depleted proteins clustered to KEGG pathways were involved in metabolism (Fig. 3B). Of proteins detected in the isolated nuclei samples, 59% were classified as nuclear by gene ontology (GO) analysis using the Database for Annotation, Visualization and Integrated Discovery (DAVID). This is nearly double the 36% classified as nuclear in whole muscle tissue samples where sarcomeric and mitochondrial proteins were the most abundant proteins detected (for a full list of identified proteins, see Table S1, Supporting information). As illustrated in Fig. 3C, nuclear proteins were dramatically enriched in nuclei samples compared to whole muscle tissue, even among relatively low enriched proteins, while proteins from other cellular compartments were depleted in all but the highest percentiles for that compartment. The cytoplasmic proteins enriched in the 90^th^ percentile were predominately keratins, which could have been introduced due to handling of the purified nuclei samples. These results establish the compatibility of the nuclear isolation procedure with downstream analysis by mass spectrometry, highlight the increased proteomic depth of nuclear proteins afforded by nuclear isolation, and represent a detailed myonuclear proteome.

### The myonuclear proteome changes with age

Given the impact that aging has on skeletal muscle function (Demontis *et al*., 2013) and gene expression (Zahn *et al*., 2006) and confident in our ability to isolate high‐purity myonuclei in quantifiable amounts, we applied this approach to investigate changes in the myonuclear proteome with aging. We isolated nuclei from GA and RF muscles of young (3‐month) and old (24‐month) mice and analyzed the samples by mass spectrometry. Muscles were pooled from two mice and five replicates were analyzed for each age. After imputation and correction for batch effect, samples were compared based on p‐value and mean fold change. As in the previous experiment, 60% of the proteins detected were classified as nuclear by GO.

**Figure 4:**
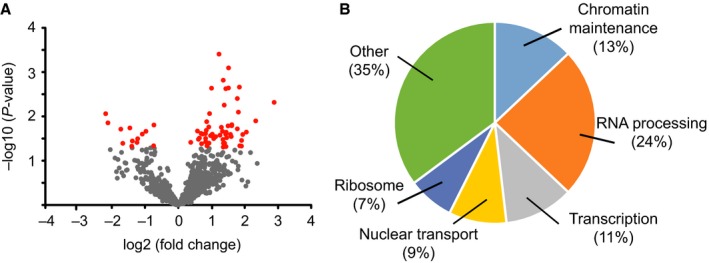
Aging of the myonuclear proteome. (A) The mean log2 fold change in LFQ (label‐free quantification) between young (3‐month) and old (24‐month) mouse myonuclei proteins was plotted against the ‐log10 *P*‐value for each protein (*n* = 5 at each age). Proteins that significantly changed with age more than 1.5‐fold (*P* < 0.05) are plotted in red: All others are plotted in gray. (B) Proteins that significantly changed with age in myonuclei were categorized by primary function and depicted as a percentage of total changed proteins.

The levels of 54 of 779 identified proteins (7%) changed significantly with age (Fig. 4A, full list in Table S2, Supporting information). Because the samples are fractionated, we cannot distinguish overall changes in steady state protein levels from a change in nuclear distribution. Of the significantly changed proteins (*P* < 0.05, fold change >1.5), 43 were more abundant in nuclei from old muscle, 1.5‐ to 7‐fold higher than young muscle levels, and 11 were less abundant, 1.5‐ to 4.5‐fold lower than young muscle levels (Table 1). The two categories with the most changed proteins were chromatin maintenance and RNA processing (Fig. 4B). The changes, both increases and decreases, in levels of proteins related to chromatin maintenance suggest general epigenetic changes with aging. In addition to age‐related increases in levels of RNA processing proteins, levels of proteins associated with transcriptional regulation increased, suggesting overall changes in transcript production and/or processing. Nuclear transport protein levels also increased, potentially indicating alterations in nuclear transport and potentially consequential mislocalization of proteins. Ribosomal protein levels also increased with age, which could be related to changes in ribosome assembly and/or altered nuclear transport or increased rough ER contribution to the aged nuclei fraction. Of the changed proteins from non‐nuclear cellular compartments, most proteins were classified as plasma membrane proteins and mitochondrial proteins. That these proteins change could reflect age‐related changes in the overall protein abundance or a differential association with young and old myonuclei. These data show that nuclear levels of markers of diverse nuclear processes including chromatin organization, RNA processing, and nuclear transport are altered during aging of mouse skeletal muscles.

**Table 1:** Myonuclear proteins that change significantly with age

log2 fold change	*P*‐value	Uniprot ID	Gene Symbol	Protein name	Role
−2.19	0.009	P43276	*Hist1h1b*	Histone H1.5	Chromatin maintenance
−2.12	0.014	Q70IV5‐2	*Synm*	Synemin	Cytoskeletal
−1.73	0.019	Q6PIC6	*Atp1a3*	Sodium/potassium‐transporting ATPase subunit alpha‐3	Membrane ion pump
−1.67	0.040	O54941	*Smarce1*	SWI/SNF‐related matrix‐associated actin‐dependent regulator of chromatin subfamily E member 1	Chromatin maintenance
−1.46	0.018	Q91XV3	*Basp1*	Brain acid soluble protein 1	Transcription
−1.40	0.047	P49813	*Tmod1*	Tropomodulin‐1	Sarcomeric
−1.38	0.036	Q9D6R2‐2	*Idh3a*	Isocitrate dehydrogenase [NAD] subunit alpha, mitochondrial	Metabolism
−1.24	0.039	P62242	*Rps8*	40S ribosomal protein S8	Ribosomal
−1.22	0.032	A2AUC9	*Klhl41*	Kelch‐like protein 41	Myofibril assembly
−1.08	0.025	Q9ESU6	*Brd4*	Bromodomain‐containing protein 4	Chromatin maintenance
−0.73	0.016	P51637	*Cav3*	Caveolin‐3	Membrane protein scaffold
0.69	0.024	Q810A7‐2	*Ddx42*	ATP‐dependent RNA helicase DDX42	RNA processing
0.70	0.041	G3UX35	*Smarca4*	Isoform of Q3TKT4, Transcription activator BRG1	Chromatin maintenance
0.82	0.031	P62908	*Rps3*	40S ribosomal protein S3	Ribosomal
0.84	0.038	Q8K4Q8	*Colec12*	Collectin‐12	Cell signaling
0.84	0.021	Q60865	*Caprin1*	Caprin‐1	RNA processing
0.86	0.013	Q64511	*Top2b*	DNA topoisomerase 2‐beta	Transcription
0.87	0.046	E9Q7Q3	*Tpm3*	Isoform of P21107, Tropomyosin alpha‐3 chain	Sarcomeric
0.93	0.040	Q8K2T8	*Paf1*	RNA polymerase II‐associated factor 1 homolog	Transcription
0.97	0.027	Q64522	*Hist2h2ab*	Histone H2A type 2‐B	Chromatin maintenance
1.00	0.002	S4R1C4	*Atp2b2*	Isoform of Q9R0K7, Calcium‐transporting ATPase	Membrane ion pump
1.03	0.025	P62309	*Snrpg*	Small nuclear ribonucleoprotein G	RNA processing
1.07	0.032	Q8BH74	*Nup107*	Nuclear pore complex protein Nup107	Nuclear Transport
1.13	0.029	Q3UN88	*Mcpt4*	Isoform of P21812, Mast cell protease 4	Cytoplasmic protease
1.26	0.026	Q922P9	*Glyr1*	Putative oxidoreductase GLYR1	Chromatin maintenance
1.35	0.033	G3UZI2	*Syncrip*	Isoform of Q7TMK9, Heterogeneous nuclear ribonucleoprotein Q	RNA processing
1.35	0.049	Q99JY0	*Hadhb*	Trifunctional enzyme subunit beta, mitochondrial	Metabolism
1.36	0.002	P08113	*Hsp90b1*	Endoplasmin	ER chaperone
1.36	0.040	Q8CJF7	*Ahctf1*	Protein ELYS	Nuclear Transport
1.38	0.006	P32067	*Ssb*	Lupus La protein homolog	RNA processing
1.40	0.040	P68040	*Gnb2l1*	Guanine nucleotide‐binding protein subunit beta‐2‐like 1	Cell signaling
1.43	0.025	P47857	*Pfkm*	ATP‐dependent 6‐phosphofructokinase, muscle type	Metabolism
1.43	0.002	Q91YQ5	*Rpn1*	Dolichyl‐diphosphooligosaccharide–protein glycosyltransferase subunit 1	ER glycosylation
1.44	0.048	Q60749	*Khdrbs1*	KH domain‐containing, RNA‐binding, signal transduction‐associated protein 1	RNA processing
1.44	0.023	O08539‐2	*Bin1*	Myc box‐dependent‐interacting protein 1	Apoptotic process
1.46	0.030	Q8QZT1	*Acat1*	Acetyl‐CoA acetyltransferase, mitochondrial	Metabolism
1.50	0.016	O35326	*Srsf5*	Serine/arginine‐rich splicing factor 5	RNA processing
1.51	0.002	P62858	*Rps28*	40S ribosomal protein S28	Ribosomal
1.52	0.001	P10852	*Slc3a2*	4F2 cell‐surface antigen heavy chain	Membrane transporter
1.57	0.025	Q9D8E6	*Rpl4*	60S ribosomal protein L4	Ribosomal
1.59	0.028	O35343	*Kpna4*	Importin subunit alpha‐3	Nuclear Transport
1.61	0.016	P42669	*Pura*	Transcriptional activator protein Pur‐alpha	Transcription
1.61	0.015	Q99MR6‐3	*Srrt*	Serrate RNA effector molecule homolog	RNA processing
1.78	0.004	A2AW05	*Ssrp1*	Isoform of Q08943, FACT complex subunit SSRP1	Transcription
1.78	0.019	E9PYL9	*Gm10036*	Protein Gm10036	RNA processing
1.81	0.008	Q8VHM5	*Hnrnpr*	Heterogeneous nuclear ribonucleoprotein R	RNA processing
1.84	0.002	P30275	*Ckmt1*	Creatine kinase U‐type, mitochondrial	Metabolism
1.85	0.046	Q6ZWY9	*Hist1h2bc*	Histone H2B type 1‐C/E/G	Chromatin maintenance
1.90	0.047	P70168	*Kpnb1*	Importin subunit beta‐1	Nuclear Transport
1.90	0.035	P62315	*Snrpd1*	Small nuclear ribonucleoprotein Sm D1	RNA processing
1.94	0.026	Q9CQF3	*Nudt21*	Cleavage and polyadenylation specificity factor subunit 5	RNA processing
2.05	0.023	D3YWX2	*Ylpm1*	Isoform of Q9R0I7, YLP motif‐containing protein 1	RNA processing
2.33	0.013	Q8VE37	*Rcc1*	Regulator of chromosome condensation	Nuclear Transport
2.89	0.005	Q99KK2	*Cmas*	N‐acylneuraminate cytidylyltransferase	Metabolism

### Myonuclei share common aging pathways with brain nuclei

**Figure 5:**
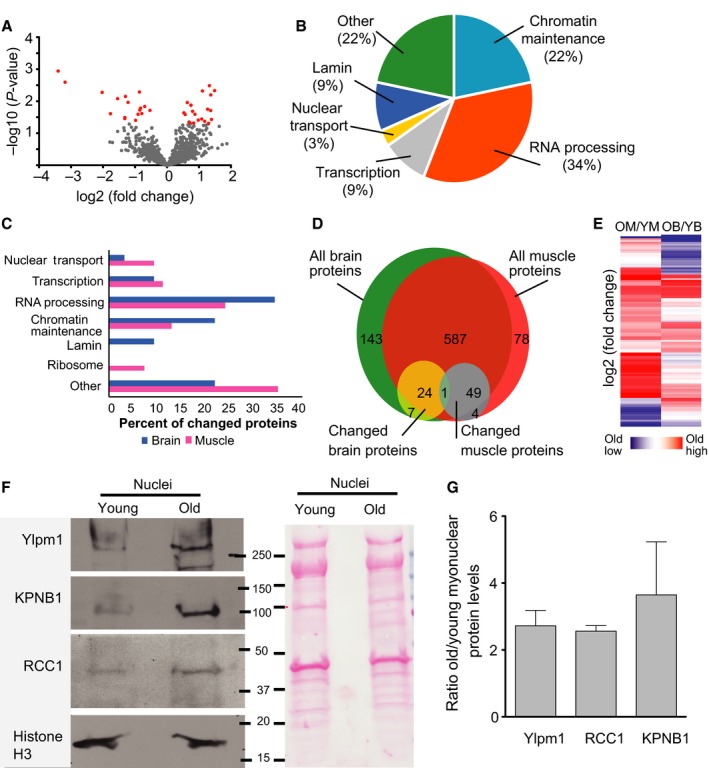
Comparison of age‐related alterations in muscle and brain nuclear proteomes (A) The mean log2 fold change in LFQ between young (3‐month) and old (24‐month) mouse brain nuclei proteins was plotted against the ‐log10 p‐value for each protein (*n* = 5 at each age). Proteins that significantly changed with age more than 1.5‐fold (*P* < 0.05) are plotted in red: all others are plotted in gray. (B) Proteins that changed with age in brain nuclei were categorized by primary function and depicted as a percentage of total changed proteins. (C) The percentage of proteins that changed with age that were assigned to each GO category was compared between brain and muscle samples. (D) The full sets of proteins detected in muscle or brain nuclear samples were compared to the subsets that significantly changed with age. The majority of proteins analyzed were detected in both tissues, but the subsets that changed with age differed greatly between tissues. Of 743 proteins detected in muscle nuclei and 811 proteins detected in brain nuclei, 661 were common to both tissues. Of the proteins that changed with age in myonuclei (54) or brain nuclei (32), only 1 was changed in both. (E) Heat map of all proteins that changed significantly with age in either tissue; aging‐related changes differ between brain and muscle nuclei. Blue indicates lower levels in old samples, and red indicates higher levels in old samples. OM = old muscle; YM = young muscle; OB = old brain; YB = young brain. (F) Representative immunoblot and accompanying Ponceau staining. The immunoblot was probed for target proteins identified as changed with age by mass spectrometry. (G) Quantification of immunoblot by densitometry (*n* = 3–4, error bar = standard error of the mean).

To investigate whether the changes to the myonuclear proteome reflect general age‐related changes or muscle‐specific changes, we compared the age‐related changes in myonuclei to those observed in brain nuclei, another excitatory, long‐lived, postmitotic tissue. We isolated and processed brain nuclei from young (3‐month) and old (24‐month) mice as described above for myonuclei. By immunoblotting, isolated brain nuclei were free of contamination from mitochondrial and cytoplasmic compartments (Fig. S2, Supporting information). Similar to the myonuclear samples, 61% of the proteins identified from brain nuclei were classified as nuclear by GO. Analysis revealed that levels of 32 of 845 identified proteins (4%) changed significantly (*P* < 0.05, >1.5‐fold change) between young and old brain nuclei (Fig. 5A). Of the changed proteins, 21 were increased in abundance in aged brain nuclei, 1.5‐ to 3‐fold compared to young brain levels, while 11 decreased, 1.5‐ to 10‐fold compared to young brain levels (Table 2). Similar to the changes detected in myonuclei with age, the majority of these proteins were related to chromatin maintenance and RNA processing (Fig. 5B).

**Table 2:** Brain nuclear proteins that change significantly with age

log2 fold change	*P*‐value	Uniprot ID	Gene Symbol	Protein name	Role
−3.40	0.001	P10854	*Hist1h2bm*	Histone H2B type 1‐M	Chromatin maintenance
−3.19	0.003	P84228	*Hist1h3b*	Histone H3.2	Chromatin maintenance
−2.03	0.005	D3Z7R4	*Syt1*	Isoform of P46096, Synaptotagmin‐1	Vesicular trafficking
−1.77	0.024	Q9CWF2	*Tubb2b*	Tubulin beta‐2B chain	Cytoskeletal
−1.54	0.008	Q8QZY9	*Sf3b4*	Splicing factor 3B subunit 4	RNA processing
−1.32	0.035	Q9QYG0	*Ndrg2*	Protein NDRG2	Signal transduction
−1.32	0.032	Q9ES97‐3	*Rtn3*	Reticulon‐3	Beta amyloid regulation
−0.86	0.005	A2AR02	*Ppig*	Peptidyl‐prolyl cis‐trans isomerase G	Lamin
−0.79	0.024	Q9QYX7‐2	*Pclo*	Protein piccolo	Cytoskeletal
−0.70	0.014	P28659‐2	*Celf1*	CUGBP Elav‐like family member 1	RNA processing
−0.53	0.019	Q0P678	*Zc3 h18*	Zinc finger CCCH domain‐containing protein 18	RNA processing
0.53	0.023	P0C0S6	*H2afz*	Histone H2A.Z	Chromatin maintenance
0.57	0.026	Q6PDM2	*Srsf1*	Serine/arginine‐rich splicing factor 1	RNA processing
0.64	0.011	P21619	*Lmnb2*	Lamin‐B2	Lamin
0.69	0.045	Q9CX86	*Hnrnpa0*	Heterogeneous nuclear ribonucleoprotein A0	RNA processing
0.71	0.046	P70372	*Elavl1*	ELAV‐like protein 1	RNA processing
0.75	0.013	Q64525	*Hist2h2bb*	Histone H2B type 2‐B	Chromatin maintenance
0.75	0.013	A0A087WRG2	*U2surp*	Isoform of Q6NV83, U2 snRNP‐associated SURP motif‐containing protein	RNA processing
0.80	0.021	P70288	*Hdac2*	Histone deacetylase 2	Chromatin maintenance
0.84	0.048	Q91VR5	*Ddx1*	ATP‐dependent RNA helicase DDX1	RNA processing
0.88	0.026	Q9CU62	*Smc1a*	Structural maintenance of chromosomes protein 1A	Chromatin maintenance
0.98	0.039	G3UZ34	*Eftud2*	Isoform of O08810, 116 kDa U5 small nuclear ribonucleoprotein component	RNA processing
1.09	0.036	Q62318	*Trim28*	Transcription intermediary factor 1‐beta	Transcription
1.11	0.005	Q9CQI7	*Snrpb2*	U2 small nuclear ribonucleoprotein B’’	RNA processing
1.18	0.042	P63158	*Hmgb1*	High mobility group protein B1	Chromatin maintenance
1.27	0.018	P10126	*Eef1a1*	Elongation factor 1‐alpha 1	Translation
1.30	0.049	Q61191	*Hcfc1*	Host cell factor 1	Transcription
1.33	0.003	P48678‐2	*Lmna*	Prelamin‐A/C	Lamin
1.37	0.019	Q8VE37	*Rcc1*	Regulator of chromosome condensation	Nuclear Transport
1.37	0.006	Q6ZPZ3‐2	*Zc3h4*	Zinc finger CCCH domain‐containing protein 4	RNA processing
1.39	0.037	Q7TNT2‐2	*Far2*	Fatty acyl‐CoA reductase 2	Metabolism
1.49	0.005	A0A0G2JD95	*Rsbn1*	Isoform of Q80T69, Round spermatid basic protein 1	Transcription

Several cellular processes changed with age in nuclei from both brain and muscle (Fig. 5C). Brain and muscle nuclei shared increases in markers of transcription, and RNA processing with age as well as overall changes in chromatin maintenance markers, supporting the concept that these are common pathways affected in aging. Two pathways were uniquely changed in nuclei from aged muscle versus brain. Nuclear lamin levels increased in aged brain nuclei but not myonuclei, while ribosomal proteins increased with age in myonuclei, but not brain nuclei. Despite the similarity in changed processes, the only protein significantly changed with age in both tissues was RCC1 (Fig. 5D), which increased more than 2‐fold in aged nuclei in both tissues. RCC1 is the major guanine nucleotide exchange factor for Ran, helping to establish the Ran GTP gradient, which is the driving force in nucleocytoplasmic transport (Izaurralde *et al*., 1997). This increase in RCC1 in both aged brain and muscle nuclei may suggest age‐related changes in nuclear transport (Fig. 5C) in both tissues.

While some of the same processes were affected by aging in both brain and muscle nuclei, the specific proteins that changed differed between the two tissues. Most of the proteins that changed in the nuclei of either tissue were detected in both tissues but only changed in one (Fig. 5D): 49 of 54 proteins (91%) that changed in muscle were detected in brain nuclei and 24 of 32 proteins (75%) that changed in brain nuclei were detected in myonuclei. Indeed, when examining the proteins that significantly change with aging in each tissue, it is clear that in individual proteins change with age quite differently in brain than in muscle (Fig. 5E). The increase in protein levels of Ylpm1, Kpnb1, and RCC1 were verified by Western blot of young and old myonuclear lysates (Fig. 5F,G). Taken together, comparison of the nuclear proteomes of muscle and brain suggests that tissue‐specific as well as common pathways, such as epigenetic regulation, RNA processing, and nuclear transport, are altered with age.

## Discussion

In this study, we present a method optimized to yield high‐purity myonuclei from relatively small samples for downstream analysis by flow cytometry, biochemistry, and mass spectrometry. We exploited this approach to interrogate the myonuclear proteome and investigate changes to the myonuclear proteome that occur with age.

Isolating nuclei from skeletal muscle has historically been difficult. Several researchers have developed approaches to isolate nuclei from skeletal muscle, each technique optimized for a downstream application like ChIP (Ohkawa *et al*., 2012), analysis of nuclear envelopes (Wilkie & Schirmer, 2008), or comparison of multiple cellular compartments (Dimauro *et al*., 2012). Our goal was to develop an isolation technique compatible with downstream analysis by flow cytometry and mass spectrometry using relatively small sample sizes to preserve biological variability. Our approach results in high‐purity myonuclei isolated from a single GA or RF muscle sufficient for analysis by flow cytometry and pooled GA and RF muscles from two mice sufficient for analysis by mass spectrometry. The isolated nuclei are impermeable to large dextrans, indicating that the nuclear envelopes remain intact, and soluble nuclear proteins such as transcription factors and RNA processing enzymes are readily detectable. Both results indicate that the nuclei do not undergo major loss of protein during purification.

Genetic labeling approaches could complement our biochemical fractionation strategy. Fluorescent labeling using nTnG transgenic mice as used here combined with fluorescent activated nuclear sorting or a recently developed approach termed isolation of nuclei tagged in specific cell types (INTACT) (Deal & Henikoff, 2011; Jankowska *et al*., 2016) could increase purity of isolated myonuclei. However, both methods require generation of new mouse strains for each cell type of interest. Thus, our biochemical isolation has the advantage of isolating a high percentage of myonuclei without genetic labeling.

Using biochemical fractionation, we dramatically enriched the nuclear proteome from skeletal muscle. In many tissues, nuclear proteins are readily detectable by proteomics in unfractionated tissue. Nuclear proteins in skeletal muscle are underrepresented because they comprise a small percentage of the overall muscle proteome (Table S3, Supporting information). Consistent with a previous report (Deshmukh *et al*., 2015), we find that >60% of the peptide reads identified by mass spectrometry of whole muscle tissue correspond to contractile proteins. Others have employed various approaches to increase proteomic detection of noncontractile proteins in skeletal muscle including depletion of contractile proteins (Carberry *et al*., 2014; Gueugneau *et al*., 2014) and enriching less detected proteins (Gannon & Ohlendieck, 2012). While these approaches have enriched some noncontractile proteins, they identified few nuclear proteins. Our study offers the first systematic examination of the myonuclear proteome, identifying 535 annotated nuclear proteins by GO, and provides tools for others to interrogate changes in this important cellular compartment.

As muscles age, muscle mass and function decrease and myofibers undergo fiber‐type switching. Proteomic studies of aging muscle have described alterations in the most abundant proteins in muscle: contractile and metabolic proteins (Baraibar *et al*., 2013). Microarray and RNAseq experiments have identified major shifts in transcript levels as muscles age (Zahn *et al*., 2006; Kim *et al*., 2014; Su *et al*., 2015). To investigate how aging affects myonuclei at the protein level, we compared the myonuclear proteomes of young and old mice. Some of the changes we detected in protein levels mirror changes in transcript levels: transcript levels of RNA‐binding proteins Hnrnpr (Welle *et al*., 2003), Srsf5, Paf1, and Ddx41 (Su *et al*., 2015), a chromatin maintenance protein Ssrp1 (Su *et al*., 2015), and a nuclear transport protein Kpnb1 (Swindell, 2009) increase with age consistent with changes we observed in protein levels. Protein levels of the RNA‐binding protein Khdrbs1 also increase with age (Laohavinij, 2015). In addition to these specific proteins, transcript levels for RNA processing machinery genes in general increase with age (Gheorghe *et al*., 2014). Similarly, we identified an increase in RNA‐binding protein levels within the nucleus with age. Our data showed an increase in ribosomal proteins in myonuclei with age. Others have characterized increases in transcript levels of ribosomal proteins (Calura *et al*., 2008; Fedorov *et al*., 2014) and decreased ribosome biogenesis (Kirby *et al*., 2015) with age. While our results could reflect increased ER contribution to the nuclear fraction from older mice, they could also support abnormalities in ribosome biogenesis or export. With our method of enriching nuclear proteins, we were able to investigate the myonuclear proteome at greater depth than previously possible and identify novel myonuclear age‐related changes.

To determine whether the age‐related changes we detected in the myonuclear proteome were common to nuclei of other excitatory postmitotic tissues, we compared aging in brain and muscle nuclei. Previous comparisons of transcript levels from whole skeletal muscle and neuronal tissues with aging predominantly identified common changes in metabolism (Capitanio *et al*., 2009; Gheorghe *et al*., 2014), chromatin maintenance, and RNA processing (Gheorghe *et al*., 2014; Su *et al*., 2015). We likewise detected age‐related changes in proteins involved in chromatin maintenance and RNA processing in nuclei from both brain and muscle. In addition, we identified changes in transcription. While the same processes were affected in nuclei from both tissues, Rcc1 was the only protein that increased with age in nuclei from both tissues. RCC1 helps establish the Ran GTP gradient necessary for nucleocyotoplasmic transport (Izaurralde *et al*., 1997). Increased Rcc1 activity increases DNA damage repair and reduces senescence, suggesting a role in aging (Cekan *et al*., 2016). While pathways were shared between nuclei from both tissues, the individual proteins that changed differed, which is consistent with a previous study in which aging affected the same pathways in two hind‐limb muscles but via different proteins (Chaves *et al*., 2013).

In summary, our approach to isolating myonuclei allowed a deeper view into the myonuclear proteome than previously possible. Muscle‐specific changes that occur with aging in nuclear proteins could offer new insight into processes contributing to age‐related muscle loss. Isolating and interrogating myonuclei will also allow investigation into nuclear processes involved in muscle growth, regeneration, and response to disease. In addition to examining changes in the proteome, one could isolate myonuclei to interrogate the nascent transcriptome and other nuclear processes difficult to detect over cytoplasmic background. Our technique will enable future studies into a heretofore underrepresented compartment of skeletal muscle.

## Experimental procedures

### Mice

Wild‐type C57BL6 mice were obtained from Charles River laboratories (Willmington, MA) and the National Institute of Aging. nTnG mice containing an allele for nuclear‐targeted tdTomato and eGFP reporter proteins (B6N.129S6‐Gt(ROSA)26Sor^tm1(CAG‐tdTomato*,‐EGFP*)Ees^/J) (Prigge *et al*., 2013) were purchased from Jackson Laboratory (Bar Harbour, ME). These mice were bred to mice expressing Cre recombinase under control of the muscle creatine kinase promoter (B6.FVB(129S4)‐Tg(Ckmm‐cre)5Khn/J) (Bruning *et al*., 1998), also purchased from Jackson Laboratory. The genotype of the offspring was determined using PCR protocols available on the Jackson Laboratory website.

All experiments were performed using tissues from 3‐ to 24‐month‐old male C57BL6 mice or 3‐ to 6‐month‐old male mice homozygous for the nTnG transgene and heterozygous for Cre recombinase. Experiments were performed in accordance with approved guidelines and ethical approval from Emory University's Institutional Animal Care and Use Committee and in compliance with the National Institutes of Health.

### Nuclear isolation

The procedure described below was extensively modified from (Wilkie & Schirmer, 2008) to achieve sufficient sample quantity and quality for analysis by flow cytometry (a single mouse GA or RF) and mass spectrometry (pooled GA and RF from two to three mice). All steps were carried out at 4 °C.

Whole brain or GA and RF muscles were dissected, minced, and suspended in 10 mL homogenization buffer 1 (10 mm HEPES, 60 mm KCL, 0.5 mm spermidine, 0.15 mm spermine, 2 mm EDTA, 0.5 mm EGTA, 300 mm sucrose, 5 mm MgCl_2_, 2 mm dithiothreitol (DTT), and 5% complete mini protease inhibitors (CMP) (Roche Diagnostics, Risch‐Rotkreuz, Switzerland)). Subsequently, muscles were homogenized with 20–25 strokes using a 15‐mL PTFE tissue grinder with clearance 0.15–0.25 mm (VWR, Radnor, PA, USA). The homogenate was filtered (40 μm) and centrifuged at 1000×*g* for 10 min, yielding a crude nuclear pellet. The pellet was resuspended in 3 mL 1.7 m sucrose with two strokes of the dounce homogenizer and loaded over a two‐step sucrose cushion: 2.8 m sucrose and 2.0 m sucrose in 50 mm HEPES, 25 mm KCl, and 5 mm MgCl_2_. The sample was centrifuged for 3 h at 186733×*g* in a SW41Ti rotor in a Beckman Optima LE‐80k ultracentrifuge. For larger preparations for proteomics, nuclei were isolated with the same gradient but centrifuged with a SW32Ti rotor in a Beckman Optima LE‐80k centrifuge at 175587×*g* for 195 min and buffers were supplemented with 1× HALT protease and phosphatase inhibitor (Thermo Scientific, Waltham, MA, USA).

After ultracentrifugation, the nuclei concentrated at the interface between the 2.0 m and 2.8 m sucrose layers were collected, diluted 1:15 with resuspension buffer (20 mm HEPES, 10 mm KCl, 1.5 mm MgCl_2_, 0.5 mm spermidine, 0.15 mm spermine, 0.2 mm EDTA, and 5% CMP (Roche Diagnostics)), and mixed thoroughly by inverting. For optimal yield, the nuclear fraction was collected into conical tubes pretreated with 1% bovine serum albumin (BSA) to prevent nuclei sticking to tube walls. The nuclei were pelleted at 3000 ×*g* for 15 min. The small clear pellet was washed in resuspension buffer and pelleted at 2000 ×*g* for 5 min. For biochemistry and mass spectrometry, the pellet was washed two more times to remove residual BSA.

To assess the integrity of isolated nuclei, a 10‐μL aliquot of nuclei was stained with 1 μg mL^−1^ DAPI and 60 μg mL^−1^ 500kD fluorescein isothiocyanate (FITC)‐conjugated dextran (Sigma‐Aldrich, St. Louis, MO, USA) and examined by fluorescence microscopy. Nuclei were scored for exclusion of the dextran by fluorescence microscopy (D'Angelo *et al*., 2009).

### Immunoblotting

GA and RF muscles were dissected and either homogenized in homogenization buffer 2 (150 mm NaCl, 1% NP‐40, 0.5% sodium deoxycholate, 0.1% sodium dodecyl sulfate, 50 mm Tris‐pH 8, 5% CMP (Roche Diagnostics)) or processed to isolate nuclei as described above. The cytoplasmic fraction was retained after the first centrifugation step. Equal protein content (Bradford, 1976) was resolved by SDS‐PAGE electrophoresis. Proteins were transferred to nitrocellulose membranes and detected with antibodies (Table S4, Supporting information) and enhanced chemiluminescence.

### Single myofiber isolation

GA muscles were dissected and processed as described previously (Pichavant & Pavlath, 2014). Briefly, muscles were enzymatically digested, single myofibers transferred to multiwell plates and fixed with paraformaldehyde.

### Flow cytometry

Isolated nuclei were incubated with antibodies (see Table S4, Supporting information for antibodies and dilutions) or appropriate isotype controls for 30 min on ice, then washed, and incubated with secondary antibodies for 30 min on ice. Nuclei were stained with 1 μg mL^−1^ DAPI immediately before analysis. After gating on DAPI^+^ nuclei, 20 000 nuclear events from each sample were analyzed for Texas Red, AF647, and eGFP fluorescence using a BD LSRII flow cytometer. Analyses of flow cytometry data were performed using FlowJo LLC, Ashland, Oregon, USA (version X 10.0.7r2).

### LC‐MS/MS analysis

Samples were processed following established protocols (Wang *et al*., 2016). For a detailed description, see supplemental methods. Briefly, 50 μg from each sample was subjected to in‐solution trypsin digest (Herskowitz *et al*., 2010). Peptide mixtures were separated by a NanoAcquity UHPLC (Waters, Milford, FA, USA) and monitored on a Orbitrap Fusion mass spectrometer (ThermoFisher Scientific, San Jose, CA, USA). The mass spectrometer cycle was programmed for “top speed acquisition” with a cycle time of 3 s.

### Label‐free proteomic quantification

For a detailed description of quantification, see supplemental methods (Data S1). Briefly, for analysis of purified myonuclei compared to whole muscle samples, raw files were searched against the mouse UniPort reference database using SEQUEST algorithm through Proteome Discoverer 2.0 platform (Thermo Scientific, Bremen, Germany). The embedded Percolator algorithm (Kall *et al*., 2007) was used to filter the peptide spectral matches to achieve a false discovery rate (FDR) of <1%. For analysis of age‐related proteomic changes in myonuclei and brain nuclei, data were analyzed using maxquant, freely distributed by J. Cox of Max Planck Institute of Biochemistry, Munich, Germany (v1.5.2.8) with Thermo Foundation 2.0 for RAW file reading capability (Cox and Mann 2008). The search engine Andromeda was used to build and search a concatenated target‐decoy mouse UniProt Knowledgebase (UniProtKB) (53 289 target sequences downloaded April 2015 for Andromeda search within MaxQuant (Cox *et al*., 2011). The label‐free quantification (LFQ) algorithm in MaxQuant (Cox *et al*., 2014; Luber *et al*., 2010) was used for protein quantitation. Imputation of missing values using Perseus (Tyanova *et al*., 2016) was followed by batch effect correction using ComBat (Johnson *et al*., 2007). After calculating fold change and p‐value for each protein, data were analyzed by GO term using DAVID (da Huang *et al*., 2009a,b) or KEGG pathway by go‐elite v1.2.5 (Zambon *et al*., 2012).

### Image acquisition

For analysis of isolated nuclei, images were obtained using an Axioplan microscope (Carl Zeiss MicroImaging, Oberkochen, Germany) with either a 0.3 NA 10× Plan‐Neofluar objective (Carl Zeiss MicroImaging) or a 0.8 NA 25× Plan‐Neofluar objective (Carl Zeiss MicroImaging) and were recorded with a camera (Carl Zeiss MicroImaging) and Scion Image 1.63 (Scion Corporation, Torrance, CA, USA) software. For analysis of single myofibers, images were obtained using a Nikon Eclipse TE2000‐U confocal microscope (Nikon, Tokyo, Japan) with a 0.50 NA Plan Fluor 20× objective (Nikon) and were recorded with a SensiCam QE (Cooke, Campbell, CA, USA) with IPlab 4.0 (Scanalytics, Fairfax, VA, USA) software. All images were assembled and equally processed using Adobe Photoshop CS4 version 11.0.2 (Adobe, San Jose, CA, USA).

### Statistical analysis

Data were analyzed for statistical significance based on fold change, *P*‐value, or *z*‐score. *z*‐score enrichment was determined using GOelite (Zambon *et al*., 2012). A *P*‐value of < 0.05 or *z*‐score of >1.96 was considered statistically significant. Relevant changes were considered >1.5‐fold.

## Funding

National Institute of General Medical Sciences, (Grant/Award Number: ‘GM008367‘) National Institute of Arthritis and Musculoskeletal and Skin Diseases, (Grant/Award Number: ‘AR062483‘, ‘AR067645‘).

## Author contributions

AAC participated in conception and design, collection and assembly of data, data analysis and interpretation, financial support, manuscript writing, and final approval of manuscript. ED and DD were involved in sample preparation, data analysis and interpretation of proteomic experiments, and final approval of manuscript. NTS carried out data analysis and interpretation of proteomic experiments, manuscript writing, and final approval of manuscript. AHC took part in conception and design, data analysis and interpretation, manuscript writing, and final approval of manuscript. GP participated in conception and design, data analysis and interpretation, financial support, manuscript writing, and final approval of manuscript.

## Conflict of interest

None declared.

For sources cited in supplemental materials see supplementary references (Data S2).

## Supporting information


**Fig. S1** Full blots of antibodies used in Figure 1.
**Fig. S2** Biochemical purity of nuclei isolated from the brain.

**Table S1** Comparison of proteins detected in whole muscle and purified myonuclei samples.

**Table S2** Comparison of protein levels detected in nuclei isolated from brain and muscle from young and old mice.

**Table S3** Summary of nuclear proteins detected in proteomic studies of aging skeletal muscle.

**Table S4** Antibodies used.

**Data S1** Methods: Detailed descriptions of LC‐MS/MS analysis and label‐free quantification.

**Data S2** Supplementary references.
