# Supplementary figures and images for: Biochemical isolation of myonuclei employed to define changes to the myonuclear proteome that occur with aging
Source: Aging Cell. 2017 May 23;16(4):738–49. doi: 10.1111/acel.12604 (PMC5506426; doi:10.1111/acel.12604)

Figure S1

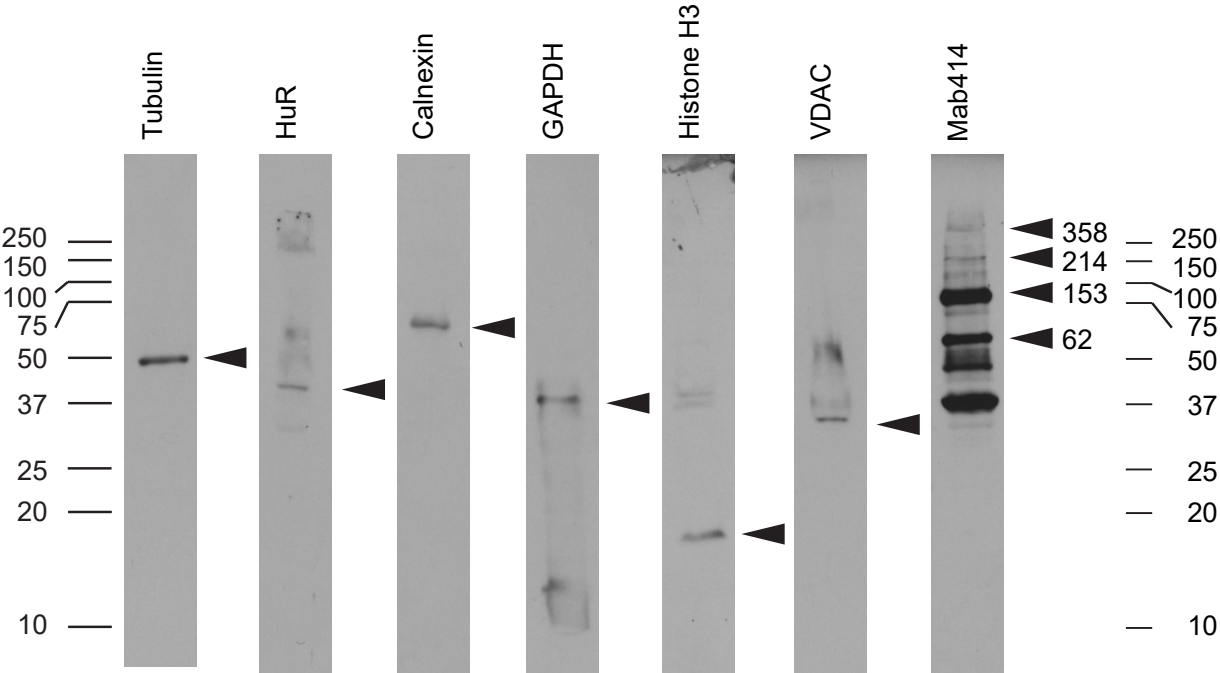

Figure S2

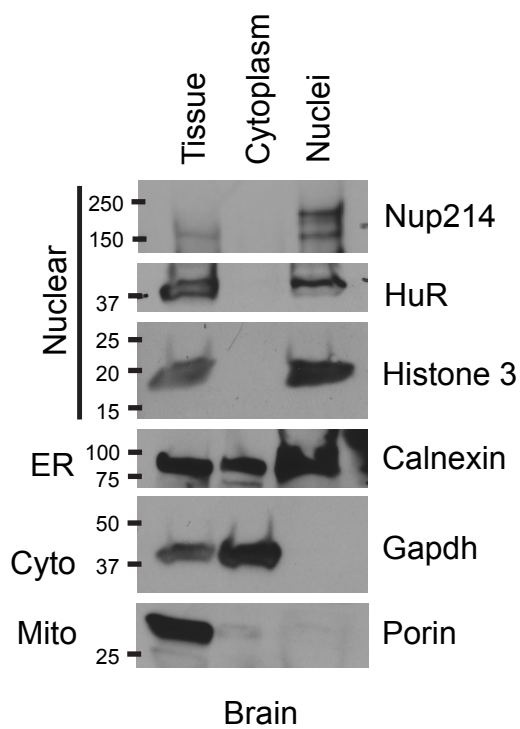

Supplement: Supplementary file 1 — Fig. S1 Full blots of antibodies used in Figure 1. Fig. S2 Biochemical purity of nuclei isolated from the brain. [file ACEL-16-738-s001.pdf]
