# Supplementary material for: Biochemical isolation of myonuclei employed to define changes to the myonuclear proteome that occur with aging
Source: Aging Cell. 2017 May 23;16(4):738–49. doi: 10.1111/acel.12604 (PMC5506426; doi:10.1111/acel.12604)
Supplement: Supplementary file 4 — Table S3 Summary of nuclear proteins detected in proteomic studies of aging skeletal muscle. [file ACEL-16-738-s004.docx]

**Supplemental Table 3:** Summary of nuclear proteins detected in proteomic studies of aging skeletal muscle

| Species | Age | Muscle | Method | Total Proteins (or spots) identified | Total nuclear proteins (by DAVID GO) | Changed proteins | Changed nuclear proteins (by DAVID GO) | Also detected in our dataset | Reference |
| --- | --- | --- | --- | --- | --- | --- | --- | --- | --- |
| Human | 56 and 78 years | vastus lateralis | 2DGE | 1919 | NR | 67 | 2 | 0 | (Gueugneau *et al.* 2014) |
| Human | 52-57 and 75-79 years | vastus lateralis | LF MS/MS | 366 | 24 | 35 | 0 | - | (Theron *et al.* 2014) |
| Human | 47-62 and 76-82 years | vastus lateralis | 2DGE | NR | NR | 19 | 0 | - | (Staunton *et al.* 2012) |
| Mouse | 12 and 24 months | gastrocnemius | LF MS/MS | 670 | 282 | 114 | 47 | 14 | (McDonagh *et al.* 2015) |
| Mouse | 6 and 27 months | gastrocnemius | mTRAQ | 236 | 81 | 53 | 8 | 0 | (Hwang *et al.* 2014) |
| Rat | 3 and 24 months | extensor digitorum longus | TMT MS/MS | 3452 | NR | 78 | 5 | 0 | (Chaves *et al.* 2013) |
| Rat | 3 and 24 months | soleus | TMT MS/MS | 1848 | NR | 174 | 49 | 0 | (Chaves *et al.* 2013) |
| Rat | 8 and 22 months | gastrocnemius | 2DGE | 3130 | NR | 83 | 15 | 0 | (Capitanio *et al.* 2009) |
| Rat | 3 and 22 months | triceps | 2DGE | NR | NR | 44 | 0 | - | (Capitanio *et al.* 2016) |
| Rat | 3 and 22 months | gastrocnemius | 2DGE | NR | NR | 87 | 0 | - | (Capitanio *et al.* 2016) |
| Rat | 3 and 30 months | gastrocnemius enriched for basic proteins | 2DGE | NR | NR | 6 | 0 | - | (Gannon & Ohlendieck 2012) |
| Rat | 3 and 30 months | gastrocnemius | 2DGE | 2493 | NR | 69 | 0 | - | (Doran *et al.* 2008) |
| Rat | 6, 18, and 27 months | gastrocnemius | TMT MS/MS | 1263 | 202 | NR | - | 26 | (Ibebunjo *et al.* 2013) |
| Rat | 3 and 26 months | gastrocnemius | 2DGE | 2865 | NR | 97 | 11 | 5 | (Donoghue *et al.* 2010) |
| Rat | 3 and 24 months | gastrocnemius | 2DGE | NR | NR | 22 | 0 | - | (Lombardi *et al.* 2009) |

Abbreviations:

DAVID GO Database for annotation visualization and integrated discovery Gene ontology

2DGE Two-dimension gel electrophoresis

LF MS/MS Label free MS/MS

TMT MS/MS Tandem mass tagged MS/MS

mTRAQ mTRAQ

NR Not reported
