# Supplementary material for: Biochemical isolation of myonuclei employed to define changes to the myonuclear proteome that occur with aging
Source: Aging Cell. 2017 May 23;16(4):738–49. doi: 10.1111/acel.12604 (PMC5506426; doi:10.1111/acel.12604)
Supplement: Supplementary file 5 — Table S4 Antibodies used. [file ACEL-16-738-s005.docx]

**Supplemental Table 4: Antibodies and dilutions used**

| Application | Antibody | Dilution | Conjugation | Source |
| --- | --- | --- | --- | --- |
| Western blot | anti-tubulin | 1:1000 |  | Sigma-Aldrich |
| Western blot | anti-histone 3 E.960.2 | 1:10000 | | Life Technologies, Carlsbad, CA |
| Western blot | anti-HuR | 1:1000 |  | Sigma-Aldrich |
| Western blot | anti-nuclear pore complex proteins (mAb 414) | 1:1000 |  | Abcam, Cambridge, UK |
| Western blot | anti-calnexin | 1:500 |  | Enzo Life Sciences, Farmingdale, NY |
| Western blot | anti-porin | 1:1000 |  | Abcam |
| Western blot | Anti-GAPDH | 1:1000 |  | Bethyl laboratories Inc |
| Western blot | anti-KPNB1 | 1:1000 |  | Bethyl laboratories Inc |
| Western blot | anti-RCC1 | 1:1000 |  | Bethyl laboratories Inc |
| Western blot | anti-ZAP3 | 1:1000 |  | Bethyl laboratories Inc |
| Western blot | anti-mouse IgG | 1:5000 | Horse radish peroxidase | Jackson ImmunoResearch Laboratories, West Grove, PA |
| Western blot | anti-rabbit IgG | 1:5000 | Horse radish peroxidase | Jackson ImmunoResearch Laboratories |
| Flow Cytometry | anti-TMEM38A | 1:250 |  | Millipore |
| Flow Cytometry | anti-nuclear pore complex proteins (mAb 414) | 1:500 |  | Abcam, Cambridge, UK |
| Flow Cytometry | anti-mouse IgG | 1:300 | Texas Red | Jackson ImmunoResearch Laboratories |
| Flow Cytometry | anti-rabbit IgG | 1:250 | Alexafluor 647 | Jackson ImmunoResearch Laboratories |
