# Supplementary material for: Biochemical isolation of myonuclei employed to define changes to the myonuclear proteome that occur with aging
Source: Aging Cell. 2017 May 23;16(4):738–49. doi: 10.1111/acel.12604 (PMC5506426; doi:10.1111/acel.12604)
Supplement: Supplementary file 6 — Data S1 Methods: Detailed descriptions of LC‐MS/MS analysis and label‐free quantification. [file ACEL-16-738-s006.docx]

**Supporting Information Listing**

Supplementary Methods: Detailed descriptions of LC-MS/MS analysis and label free quantification.

Table S1. Comparison of proteins detected in whole muscle and purified myonuclei samples.

Table S2: Comparison of protein levels detected in nuclei isolated from brain and muscle from young and old mice.

Table S3: Summary of nuclear proteins detected in proteomic studies of aging skeletal muscle

Table S4: Antibodies used

Figure S1: Full blots of antibodies used in figure 1

Figure S2: Biochemical purity of nuclei isolated from the brain

Supplementary References
