# Supplementary material for: Biochemical isolation of myonuclei employed to define changes to the myonuclear proteome that occur with aging
Source: Aging Cell. 2017 May 23;16(4):738–49. doi: 10.1111/acel.12604 (PMC5506426; doi:10.1111/acel.12604)
Supplement: Supplementary file 7 — Data S2 Supplementary references. [file ACEL-16-738-s007.docx]

**Supplemental References**

Capitanio D, Vasso M, De Palma S, Fania C, Torretta E, Cammarata FP, Magnaghi V, Procacci P, Gelfi C (2016). Specific protein changes contribute to the differential muscle mass loss during ageing. *Proteomics*. **16**, 645-656.

Capitanio D, Vasso M, Fania C, Moriggi M, Vigano A, Procacci P, Magnaghi V, Gelfi C (2009). Comparative proteomic profile of rat sciatic nerve and gastrocnemius muscle tissues in ageing by 2-D DIGE. *Proteomics*. **9**, 2004-2020.

Chaves DF, Carvalho PC, Lima DB, Nicastro H, Lorenzeti FM, Siqueira-Filho M, Hirabara SM, Alves PH, Moresco JJ, Yates JR, 3rd, Lancha AH, Jr. (2013). Comparative proteomic analysis of the aging soleus and extensor digitorum longus rat muscles using TMT labeling and mass spectrometry. *J Proteome Res*. **12**, 4532-4546.

Donoghue P, Staunton L, Mullen E, Manning G, Ohlendieck K (2010). DIGE analysis of rat skeletal muscle proteins using nonionic detergent phase extraction of young adult versus aged gastrocnemius tissue. *Journal of Proteomics*. **73**, 1441-1453.

Doran P, O'Connell K, Gannon J, Kavanagh M, Ohlendieck K (2008). Opposite pathobiochemical fate of pyruvate kinase and adenylate kinase in aged rat skeletal muscle as revealed by proteomic DIGE analysis. *Proteomics*. **8**, 364-377.

Gannon J, Ohlendieck K (2012). Subproteomic analysis of basic proteins in aged skeletal muscle following offgel pre-fractionation. *Molecular medicine reports*. **5**, 993-1000.

Gueugneau M, Coudy-Gandilhon C, Gourbeyre O, Chambon C, Combaret L, Polge C, Taillandier D, Attaix D, Friguet B, Maier AB, Butler-Browne G, Bechet D (2014). Proteomics of muscle chronological ageing in post-menopausal women. *BMC Genomics*. **15**, 1165.

Herskowitz JH, Seyfried NT, Duong DM, Xia Q, Rees HD, Gearing M, Peng J, Lah JJ, Levey AI (2010). Phosphoproteomic analysis reveals site-specific changes in GFAP and NDRG2 phosphorylation in frontotemporal lobar degeneration. *J Proteome Res*. **9**, 6368-6379.

Huang da W, Sherman BT, Lempicki RA (2009a). Bioinformatics enrichment tools: paths toward the comprehensive functional analysis of large gene lists. *Nucleic acids research*. **37**, 1-13.

Huang da W, Sherman BT, Lempicki RA (2009b). Systematic and integrative analysis of large gene lists using DAVID bioinformatics resources. *Nature protocols*. **4**, 44-57.

Hwang CY, Kim K, Choi JY, Bahn YJ, Lee SM, Kim YK, Lee C, Kwon KS (2014). Quantitative proteome analysis of age-related changes in mouse gastrocnemius muscle using mTRAQ. *Proteomics*. **14**, 121-132.

Ibebunjo C, Chick JM, Kendall T, Eash JK, Li C, Zhang Y, Vickers C, Wu Z, Clarke BA, Shi J, Cruz J, Fournier B, Brachat S, Gutzwiller S, Ma Q, Markovits J, Broome M, Steinkrauss M, Skuba E, Galarneau J-R, Gygi SP, Glass DJ (2013). Genomic and Proteomic Profiling Reveals Reduced Mitochondrial Function and Disruption of the Neuromuscular Junction Driving Rat Sarcopenia. *Molecular and Cellular Biology*. **33**, 194-212.

Johnson WE, Li C, Rabinovic A (2007). Adjusting batch effects in microarray expression data using empirical Bayes methods. *Biostatistics (Oxford, England)*. **8**, 118-127.

Kall L, Canterbury JD, Weston J, Noble WS, MacCoss MJ (2007). Semi-supervised learning for peptide identification from shotgun proteomics datasets. *Nat Meth*. **4**, 923-925.

Lombardi A, Silvestri E, Cioffi F, Senese R, Lanni A, Goglia F, de Lange P, Moreno M (2009). Defining the transcriptomic and proteomic profiles of rat ageing skeletal muscle by the use of a cDNA array, 2D- and Blue native-PAGE approach. *Journal of Proteomics*. **72**, 708-721.

McDonagh B, Sakellariou GK, Smith NT, Brownridge P, Jackson MJ (2015). Redox proteomic analysis of the gastrocnemius muscle from adult and old mice. *Data in brief*. **4**, 344-348.

Smith PK, Krohn RI, Hermanson GT, Mallia AK, Gartner FH, Provenzano MD, Fujimoto EK, Goeke NM, Olson BJ, Klenk DC (1985). Measurement of protein using bicinchoninic acid. *Anal Biochem*. **150**, 76-85.

Staunton L, Zweyer M, Swandulla D, Ohlendieck K (2012). Mass spectrometry-based proteomic analysis of middle-aged vs. aged vastus lateralis reveals increased levels of carbonic anhydrase isoform 3 in senescent human skeletal muscle. *International journal of molecular medicine*. **30**, 723-733.

Theron L, Gueugneau M, Coudy C, Viala D, Bijlsma A, Butler-Browne G, Maier A, Bechet D, Chambon C (2014). Label-free quantitative protein profiling of vastus lateralis muscle during human aging. *Mol Cell Proteomics*. **13**, 283-294.

Tyanova S, Temu T, Sinitcyn P, Carlson A, Hein MY, Geiger T, Mann M, Cox J (2016). The Perseus computational platform for comprehensive analysis of (prote)omics data. *Nat Meth*. **advance online publication**.

Zambon AC, Gaj S, Ho I, Hanspers K, Vranizan K, Evelo CT, Conklin BR, Pico AR, Salomonis N (2012). GO-Elite: a flexible solution for pathway and ontology over-representation. *Bioinformatics (Oxford, England)*. **28**, 2209-2210.
