# Supplementary material for: Biochemical isolation of myonuclei employed to define changes to the myonuclear proteome that occur with aging
Source: Aging Cell. 2017 May 23;16(4):738–49. doi: 10.1111/acel.12604 (PMC5506426; doi:10.1111/acel.12604)
Supplement: Supplementary file 8 [file ACEL-16-738-s008.docx]

**Figure S1. Full blots of antibodies used in Figure 1.** Fifty µg of whole muscle lysate was resolved by SDS-PAGE electrophoresis. The entire lane was probed with a given primary antibody and corresponding secondary antibody. The band corresponding to the protein of interest is indicated by an arrow head. Mab414 is a pan nuclear pore protein which detects multiple nuclear pore proteins (Nup) containing an FG repeat, each major Nup is identified with an arrow head and the Nup number.

**Figure S2. Biochemical purity of nuclei isolated from brain.** Nuclei were compared to total tissue and cytoplasmic fractions. Nuclei were enriched for markers of the nuclear envelope (Nup 214), RNA binding proteins (HuR), and chromatin (Histone 3) as well as endoplasmic reticulum markers (ER). Purified nuclei were depleted of cytoplasmic (Cyto) and mitochondrial (Mito) markers.
